# *COMMD7* is correlated with a novel NF-κB positive feedback loop in hepatocellular carcinoma

**DOI:** 10.18632/oncotarget.9047

**Published:** 2016-04-27

**Authors:** Lu Zheng, Chang-Lin Deng, Liang Wang, Xiao-Bing Huang, Nan You, Yi-Chen Tang, Ke Wu, Ping Liang, Na Mi, Jing Li

**Affiliations:** ^1^ Department of Hepatobiliary Surgery, Xinqiao Hospital of Third Military Medical University, Chongqing, 400037, China

**Keywords:** COMMD7, nuclear factor-kappa B, proliferation, apoptosis, hepatocellular carcinoma

## Abstract

The correlation between nuclear factor-kappa B (NF-κB) and *COMMD7* in hepatocellular carcinoma (HCC) development remained unclear. Here, our clinicopathological data showed that *COMMD7* is overexpressed in HCC with a correlation to NF-κB. Using HepG2 and SMMC-7721 cells that aberrantly overexpressed *COMMD7*, we found that NF-κB directly binds with *COMMD7* promoter and serves as an activator for *COMMD7* transcription by luciferase reporter assay, chromatin immunoprecipitation (ChIP), and electrophoretic mobility shift assay (EMSA). In both HepG2 cells and SMMC-7721 cells, the silencing of *COMMD7* significantly inhibited the cell proliferation, whereas NF-κB silencing inhibited the expression of *COMMD7* and further inhibited cell proliferation. In addition, cell apoptosis was promoted by *COMMD7* silencing, and further promoted by NF-κB silencing. Cell migration and invasion were also inhibited by *COMMD7* silencing, and further inhibited by NF-κB silencing. Thus, *COMMD7* is correlated with a novel NF-κB positive feedback loop in hepatocellular carcinoma. Developing strategies for the treatment of HCC should consider the correlation between NF-κB and *COMMD7*, so as to improve the specificity and sensitivity of therapy and to reduce toxicity.

## INTRODUCTION

Hepatocellular carcinoma (HCC) is one of the most common malignancies and is the third leading cause of cancer-related death worldwide [1–4]. HCC incidence in China is anticipated to continue to increase. HCC is the second leading cause of cancer-related deaths in China, and its mortality rate in China ranks first in the world. Currently, half of the patients with HCC are diagnosed at a late stage when conditions are unsuitable for surgical resection and liver transplantation. Meanwhile, the remaining cases of HCC are diagnosed at an early stage but most do not receive curative therapy [1–4]. Epidemiologic and clinical studies have identified many risk factors that affect HCC, including the overexpression of oncogene and low expression of tumor suppressors. However, the molecular mechanisms underlying the development of HCC, especially the correlations between genes and their functions have not yet been completely elucidated. Here, we aimed to investigate the correlation between nuclear factor-kappa B (NF-κB) and *COMMD7* in hepatocellular carcinoma.

NF-κB regulates cell survival, differentiation, and inflammation, and plays important roles in cancer, inflammatory diseases and development of the immune system [5–7]. It was demonstrated that NEMO, a crucial regulator of the NF-κB pathway, interacted with *COMMD7* [8]. *COMMD7* is a member of the *COMMD* family, which is overexpressed in HCC [9] and associated with tumor growth and invasion [10]. Silencing of *COMMD7* inhibited human HepG2 cell growth [11]. Endogenous depletion of *COMMD7* results in stabilization of *p65* (Ser468) and greater nuclear accumulation of *p65* following TNF-α stimulation [8]. *COMMD7* interacts with COMMD1 and together they down-regulate NF-κB activity [8]. In *COMMD7* silenced HepG2 cells, the inhibition rate of NF-κB was 75%, suggesting that *COMMD7* regulates the nuclear translocation of NF-κB and the consequent gene transcriptions involved in HCC growth [10]. However, it was demonstrated that mutational changes in individual proteins can cause fundamental functional changes well beyond the pathway they function in by a positive feedback loop embedded in the crosstalk [12–15]. Interestingly, many oncogenes associated with tumor development and progression are activated by NF-κB [16–18]. It is unclear whether NF-κB is also an upstream signaling factor of *COMMD7*, and it is unclear whether there is crosstalk between NF-κB and *COMMD7* that causes an oncogenic positive feedback loop.

Here, we examined the expression of *COMMD7* and NF-κB in patients with hepatocellular carcinoma, and analyzed the correlation between *COMMD7* and NF-κB. Then, we detected the expressions of *COMMD7* in human hepatocellular carcinoma cell lines and found that it was significantly upregulated inHepG2 and SMMC-7721 cells. Using these cell lines, the interaction of NF-κB and *COMMD7* promoter was further assessed by luciferase reporter assay, chromatin immunoprecipitation (ChIP), and electrophoretic mobility shift assays (EMSA). Finally, the roles of NF-κB and *COMMD7* in cell proliferation, cell apoptosis, migration and invasion of hepatocellular carcinoma cell lines, and the tumor growth were studied. We found that NF-κB correlated with the expression of *COMMD7*, playing important roles in the development of HCC. Therapeutic strategies for HCC should be explored based on the correlation between NF-κB and *COMMD7*.

## RESULTS

### Expression of *COMMD7* is up-regulated in hepatocellular carcinoma tissues

**Figure 1:**
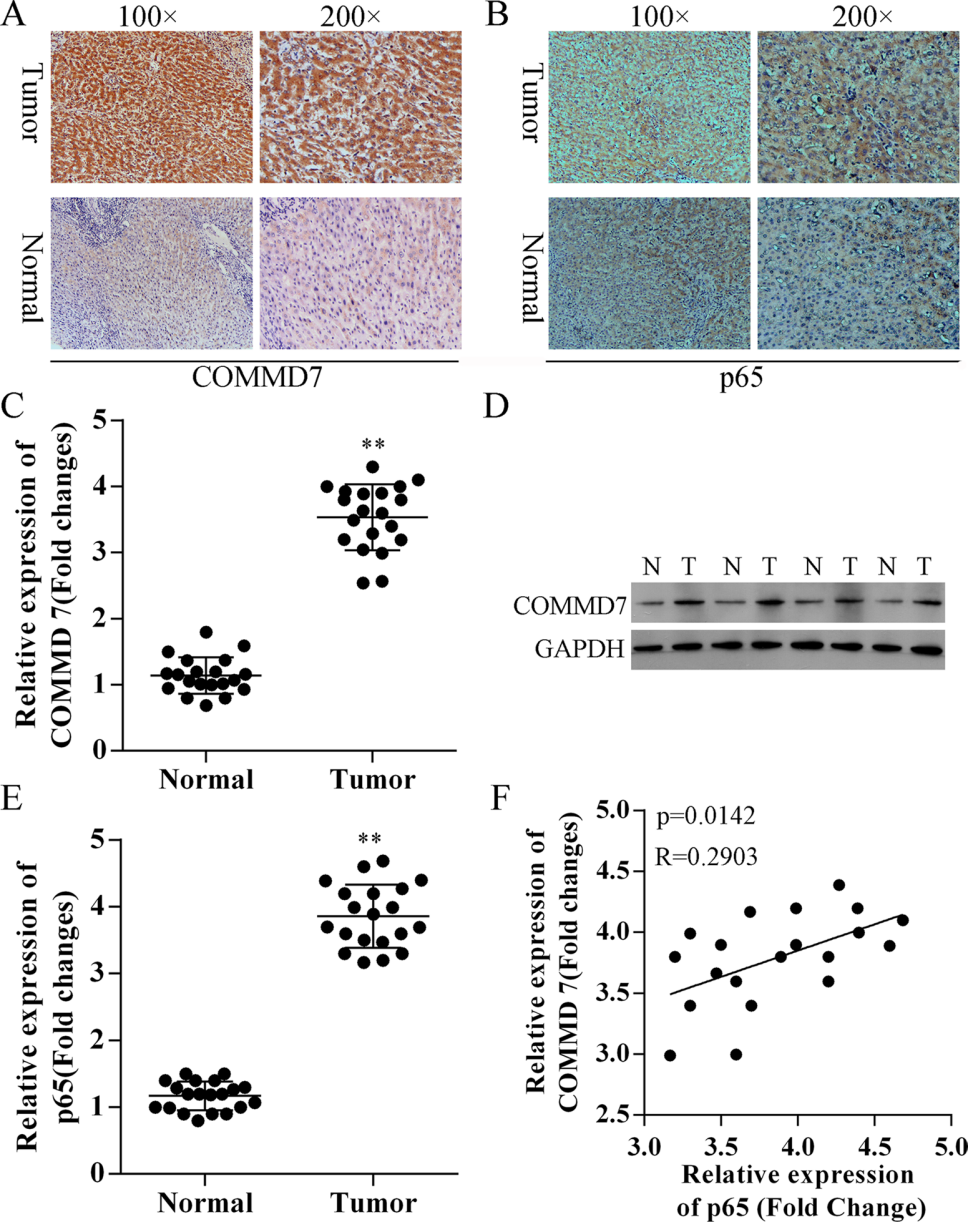
Correction between *COMMD7* and NF-κB in hepatocellular carcinoma (**A**) *COMMD7* and (**B**) *p65* expression in hepatocellular carcinoma and para-carcinoma tissues using immunohistochemistry (IHC). Representative images at different magnifications from independent experiments are shown. Tumor: hepatocellular carcinoma; Normal: para-carcinoma tissue. (**C**) qRT-PCR and (**D**) Western Blot assays revealed the up-regulation of *COMMD7* in hepatocellular carcinoma (tumor, T), compared with para-carcinoma tissues (normal, N). (**E**) qRT-PCR also revealed the up-regulation of NF-κB in hepatocellular carcinoma. (**F**) Correlation analysis revealed a significant correlation between *COMMD7* and the expression of *p65* subunit of NF-κB. **p < 0.01 vs. normal.

To investigate the role of *COMMD7* in hepatocellular carcinoma, we compared the difference in expression of *COMMD7* between hepatocellular carcinoma and para-carcinoma tissues using immunohistochemistry (IHC) (Figure 1A), qRT-PCR (Figure 1C) and Western Blot assays (Figure 1D). As shown in Figure 1A and 1B, IHC revealed that the staining of *COMMD7* and *p65* were differentially distributed between hepatocellular carcinoma and para-carcinoma tissues. The expression levels of *COMMD7* inhepatocellular carcinoma were much stronger than the para-carcinoma tissues, which suggests that *COMMD7* is involved in the pathological changes of hepatocellular carcinoma.

Based on these results, qRT-PCR and western blot were subsequently performed to further validate these findings (Figure 1C and 1D). Moreover, as shown in Figure 1E, low expression of the *p65* subunit of NF-κB in para-carcinoma tissues was also observed. Correlation analysis revealed a significant correlation between *COMMD7* and the expression of *p65* (Figure 1F).

### Expression of *COMMD7* is up-regulated in hepatocellular carcinoma cells

**Figure 2:**
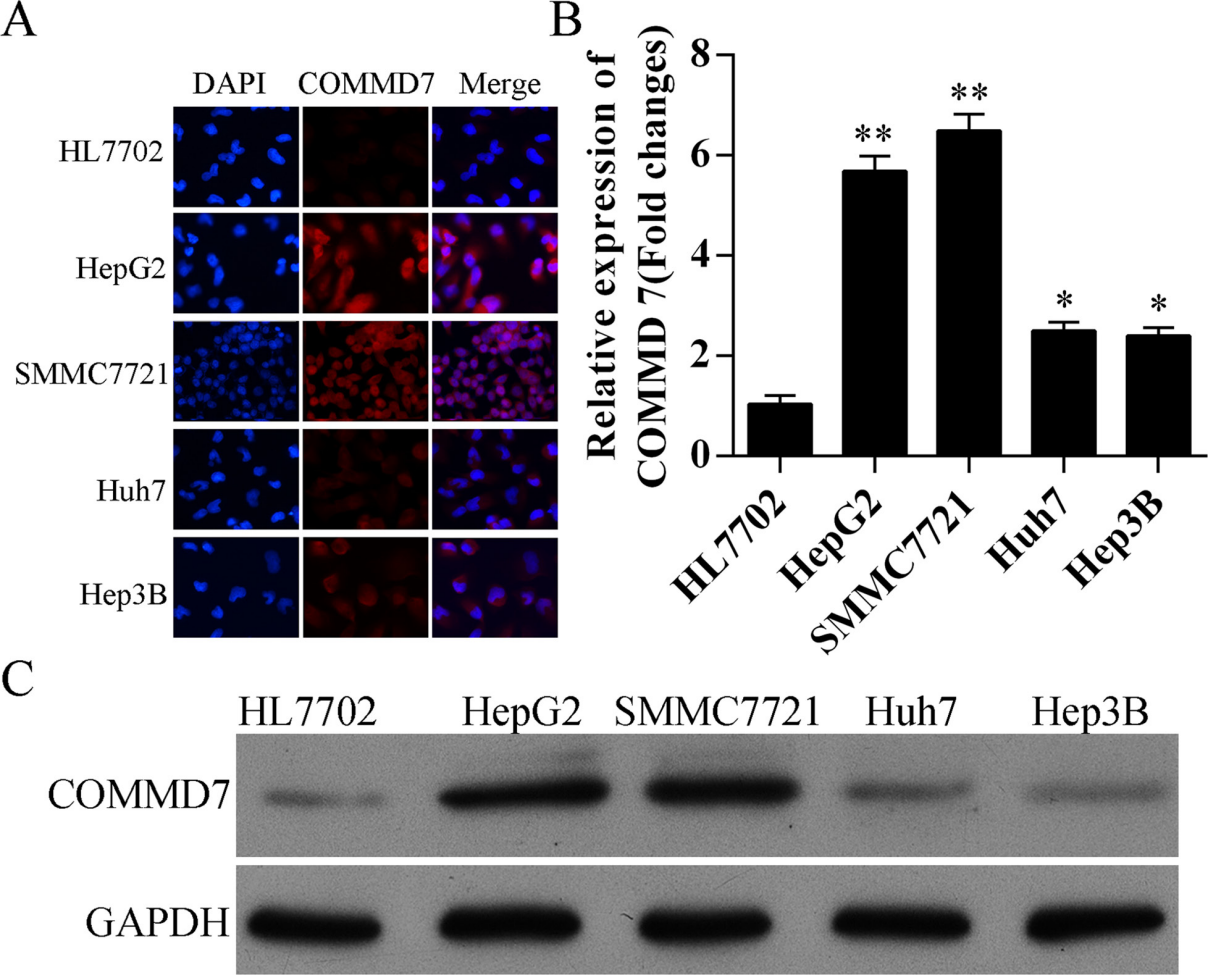
Expression of *COMMD7* is also up-regulated in hepatocellular carcinoma cells (**A**) Immunofluorescence of *COMMD7* and (**B**) expression of *COMMD7* mRNA levels in HL7702 human liver cells, and HepG2, SMMC-7721, Huh7 and Hep3B human hepatocellular carcinoma cells were detected by qRT-PCR, and (**C**) protein level was detected by Western Blot, respectively. *p < 0.05; **p < 0.01 vs. HL7702.

The expression of COMMD7 was detected by immunofluorescence, qRT-PCR, and western blot. Compared to HL7702 human liver cells, HepG2, SMMC-7721, Huh7 and Hep3B human hepatocellular carcinoma cells expressed higher level of *COMMD7* (Figure 2A), and *COMMD7* mRNA (Figure 2B). Moreover, the protein levels of *COMMD7* in HepG2 and SMMC-7721 cells were higher than Huh7 and Hep3B cells, and all of them were higher than that in HL7702 cells (Figure 2A and 2C). HepG2 and SMMC7721 cells were identified by double staining with CD34 and p65 (Supplementary Figure S2). Then, HepG2 and SMMC-7721 cells were used as cell models to investigate the role of *COMMD7* in hepatocellular carcinoma.

### NF-κB directly binds to the *COMMD7* promoter

**Figure 3:**
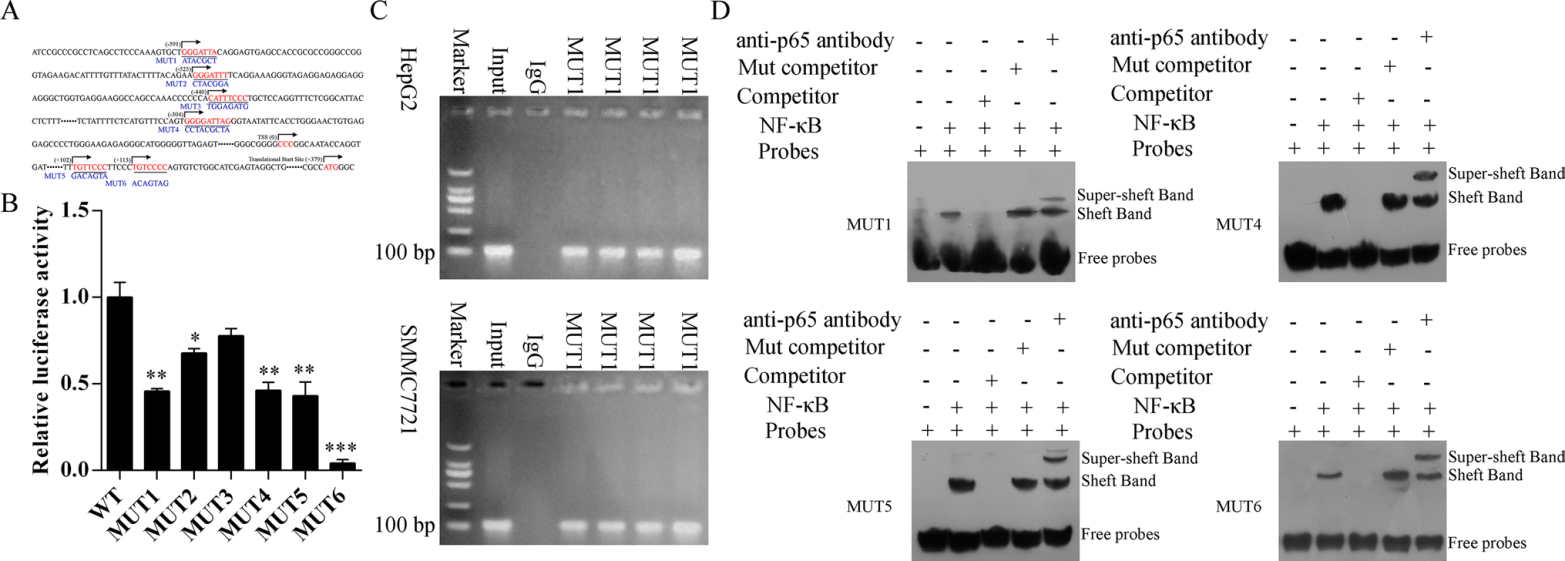
NF-κB binds to the endogenous COMMD7 promoters (**A**) Oligonucleotide sequences corresponding to the potential *COMMD7* promoters are shown, with distance from the transcription start site (TSS) indicated. Arrows denote site orientation. (**B**) 293T cells were transfected with the indicated reporter plasmids and co-transfected with NF-κB-expressing or empty pcDNA3 plasmids. Mean values (*n* = 3) of luciferase reporter activity ± standard deviation are shown. *p < 0.05; **p < 0.01 mutation (MUT) vs. wide-type (WT). (**C**) Binding of NF-κB to the endogenous *COMMD7* promoters. Chromatin-immunoprecipitated NF-κB-binding non-enriched (rabbit IgG) DNA, enriched (NF-κB) DNA, and 0.01% whole cell extract DNA in HepG2 or SMMC-7721 were amplified by PCR. (**D**) Electrophoretic mobility shift assay (EMSA). ^32^P-labeled oligonucleotide probes corresponding to the *COMMD7* promoter 1, 4, 5 and 6, complexed with NF-κB in the presence or absence of anti-NF-κB antibody or specific/mutant competitors were detected by EMSA.

We prepared oligonucleotide probes for the putative NF-κB binding regions (Figure 3A). Mutations (MUT1, 2, 4, 5 and 6) in the *COMMD7* motif significantly diminished the luciferase activity by *COMMD7* in 293T cells (*p* < 0.05, MUT2 vs. WT; *p* < 0.01, MUT1, 4, and 5 vs. WT; *p* < 0.001, MUT6 vs. WT) (Figure 3B).

To further determine whether NF-κB binds to the endogenous *COMMD7* promoters, we performed ChIP assay using HepG2 and SMMC-7721 cells (Figure 3C). Consistent with the above results, we found that NF-κB was bound to this region in both HepG2 and SMMC-7721 cells. NF-κB was bound to the *COMMD7* promoter containing putative NF-κB binding motifs in both HepG2 and SMMC-7721 cells.

Oligonucleotide probes with mutations of the *COMMD7* motif sites were synthesized and EMSAs were performed (Figure 3D). Upon incubating the *COMMD7*-promoter 1, 4, 5 and 6 oligonucleotide probes with recombinant NF-κB protein, we detected the binding of NF-κB to these sites (lane 2). Addition of specific competitor of *p65* abolished the binding (lane 3), but incubation with mutant competitor increased the putative bands (lane 4). Interestingly, addition of anti-*p65* antibody reduced the intensity of those bands, with an upward super-shift observed (lane 5). Taken together, these results suggest that *COMMD7* is a NF-κB target gene in hepatocellular carcinoma cells, and NF-κB directly correlates with *COMMD7*.

### Stable transfection of NF-κB shRNA or *COMMD7* shRNA suppresses the proliferation of hepatocellular carcinoma cells

**Figure 4:**
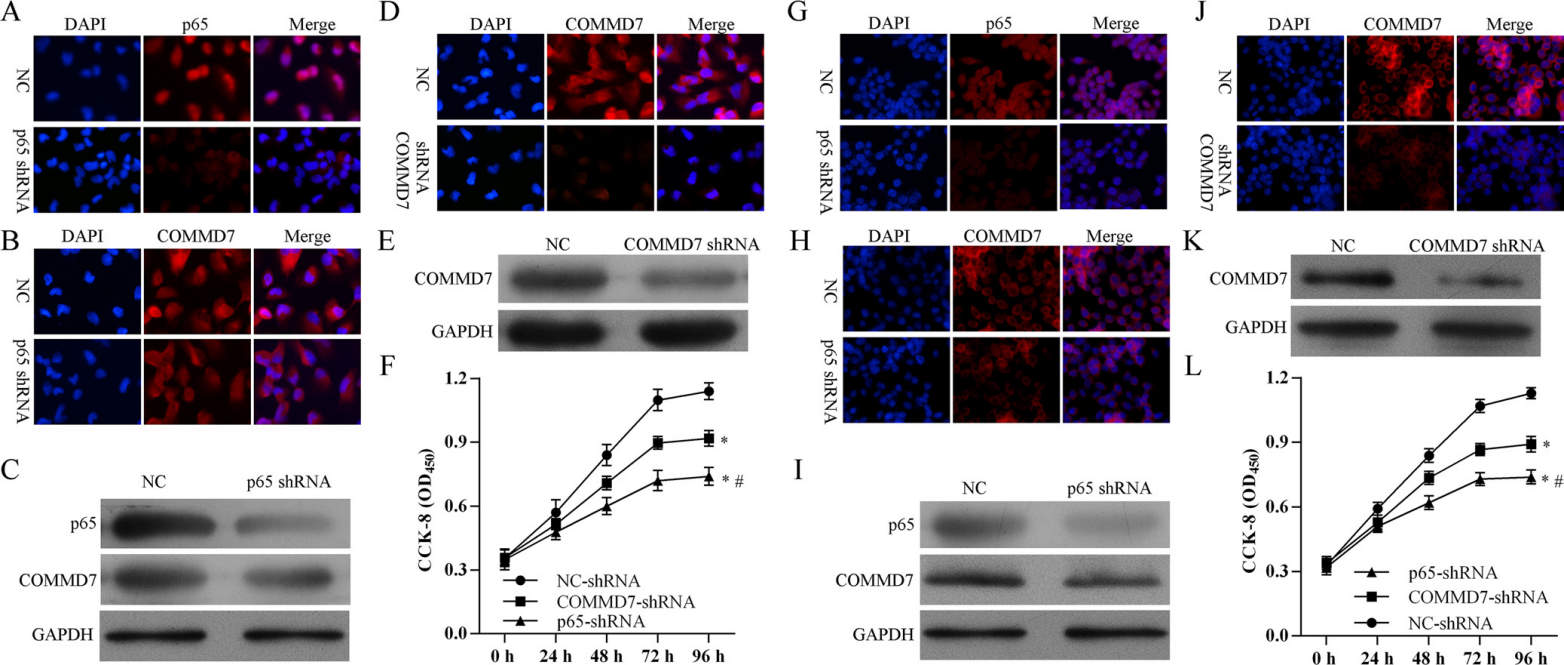
Stable transfection of NF-κB shRNA or *COMMD7* shRNA inhibited the proliferation of hepatocellular carcinoma cells (**A**–**F**): HepG2; (**G**–**L**): SMMC-7721 cells. The expression of *p65* subunit of NF-κB and *COMMD7* were detected by confocal microscopy and western blot. Expression of *p65* subunit (A, C and G, I) of NF-κB and *COMMD7* (B, C and H, I) after NF-κB shRNA transfection. Expression of *COMMD7* (D, E and J, K) after *COMMD7* shRNA transfection. (F and L) Cell proliferation was detected by MTT assay. *p < 0.05, vs. NC-shRNA; ^#^*p* < 0.05, vs. *COMMD7*-shRNA.

After stable transfection, the expression of *p65* subunit of NF-κB and *COMMD7* were detected by confocal microscopy and western blot. Stable transfection of NF-κB shRNA in HepG2 cells significantly down-regulated the expression of *p65* subunit of NF-κB and the expression of *COMMD7* (Figure 4A–4C). Transfection of *COMMD7* shRNA in HepG2 cells significantly down-regulated the expression of *COMMD7* (Figure 4D–4E). Knockdown of *COMMD7* significantly reduced the proliferation of HepG2, and knockdown of NF-κB further attenuated the proliferation (Figure 4F). In agreement with this conclusion, in SMMC-7721 cells, NF-κB shRNA also significantly down-regulated the expression of *p65* and *COMMD7* (Figure 4G–4I), and *COMMD7* shRNA significantly down-regulated the expression of *COMMD7* (Figure 4J and 4K). The cell proliferation of SMMC-7721 was reduced by *COMMD7* shRNA, and further attenuated by NF-κB shRNA (Figure 4L).

### Stable transfection of NF-κB shRNA or *COMMD7* shRNA enhances the apoptosis of hepatocellular carcinoma cells

**Figure 5:**
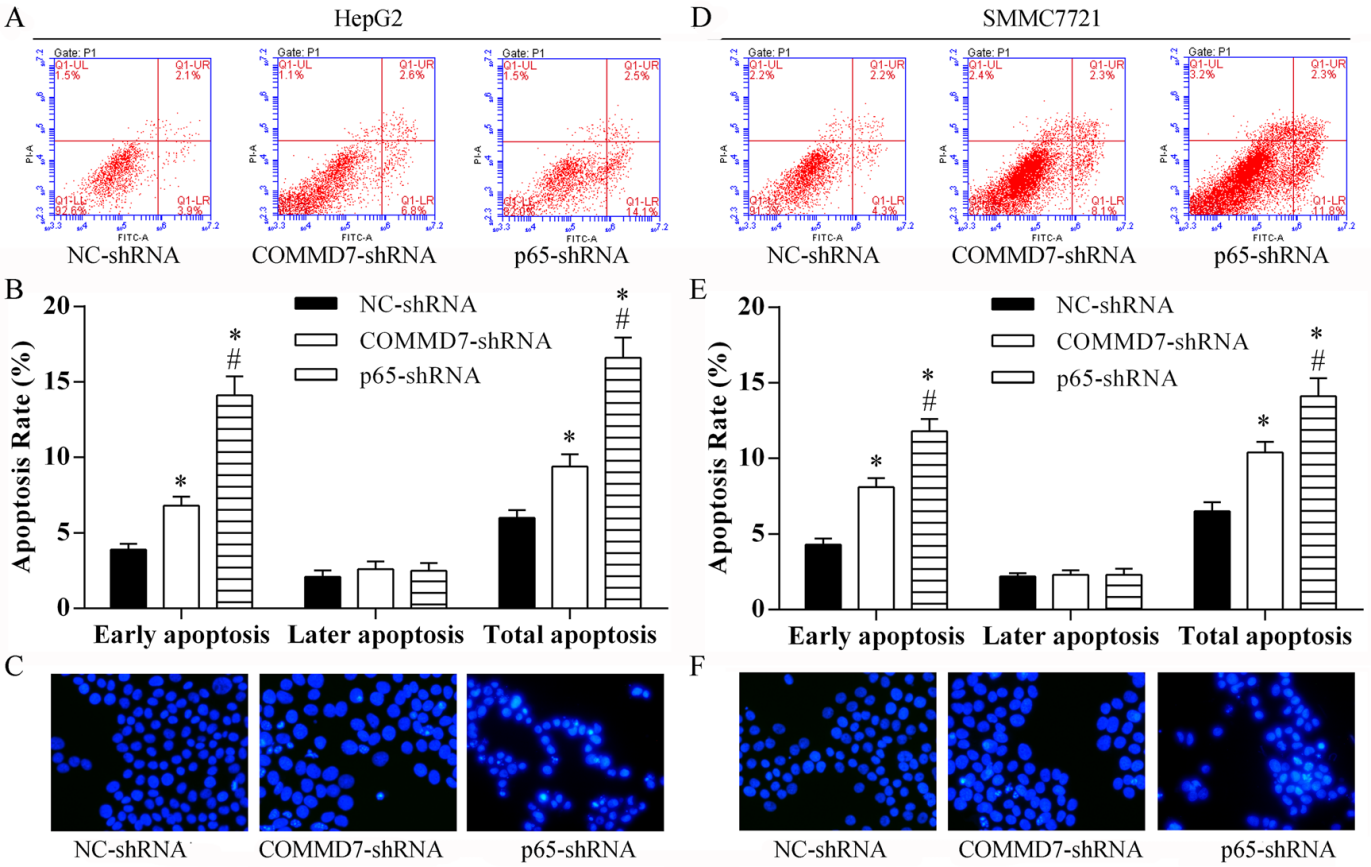
Stable transfection of NF-κB shRNA or *COMMD7* shRNA enhanced apoptosis of hepatocellular carcinoma cells (**A**–**C**): HepG2; (**D**–**F**): SMMC-7721 cells. (A and D) Cell apoptosis in each group was determined using the Annexin V-FITC/PI flow cytometry, and (B and E) proportion of apoptosis cells was measured. *p < 0.05, vs. NC-shRNA; ^#^*p* < 0.05, vs. *COMMD7*-shRNA. (C and F) The roles of COMMD7 and NF-κB in cell apoptosis were further confirmed by Hoechest staining.

Following transfection of HepG2 cells with *COMMD7* shRNA we found that cell apoptosis, especially the early apoptosis was enhanced, as detected by flow cytometry (Figure 5A and 5B). Knockdown of NF-κB showed a significant increase in early apoptosis in HepG2 cells, compared to cells transfected with *COMMD7* shRNA (Figure 5A and 5B). Similar to HepG2 cells, in SMMC-7721 cells, the total apoptosis and early apoptosis of SMMC-7721 cells were increased by *COMMD7* shRNA, and further enhanced by NF-κB shRNA (Figure 5D and 5E). The prosurvival roles of COMMD7 and NF-κB were further confirmed by Hoechest staining (Figure 5C and 5F).

### Stable transfection of NF-κB shRNA or *COMMD7* shRNA abolishes the invasion of hepatocellular carcinoma cells

**Figure 6:**
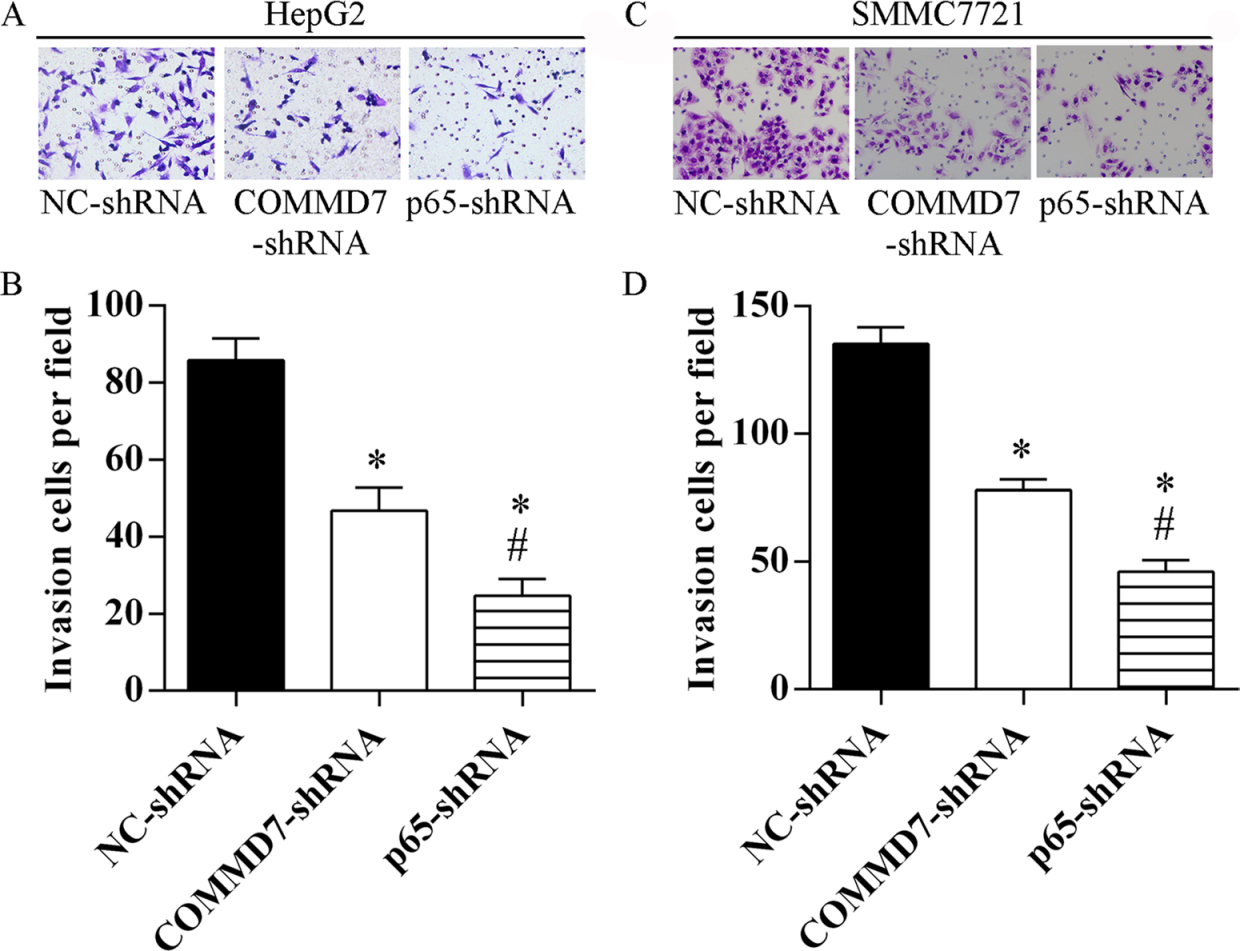
Stable transfection of NF-κB shRNA or *COMMD7* shRNA suppressed the invasion of hepatocellular carcinoma cells (**A** and **B**): HepG2; (**C** and **D**): SMMC-7721 cells. (A and C) Invasion assay was assessed by transwell chamber and migrated cells were counted (B and D). *p < 0.05, vs. NC-shRNA; ^#^*p* < 0.05, vs. *COMMD7*-shRNA.

The invasion of HepG2 (Figure 6A and 6B) and SMMC-7721 cells (Figure 6C and 6D) was significantly abolished by *COMMD7*-shRNA, and further abolished by NF-κB shRNA, respectively.

### Stable transfection of NF-κB shRNA or *COMMD7* shRNA suppresses tumorigenicity *in vivo*

**Figure 7:**
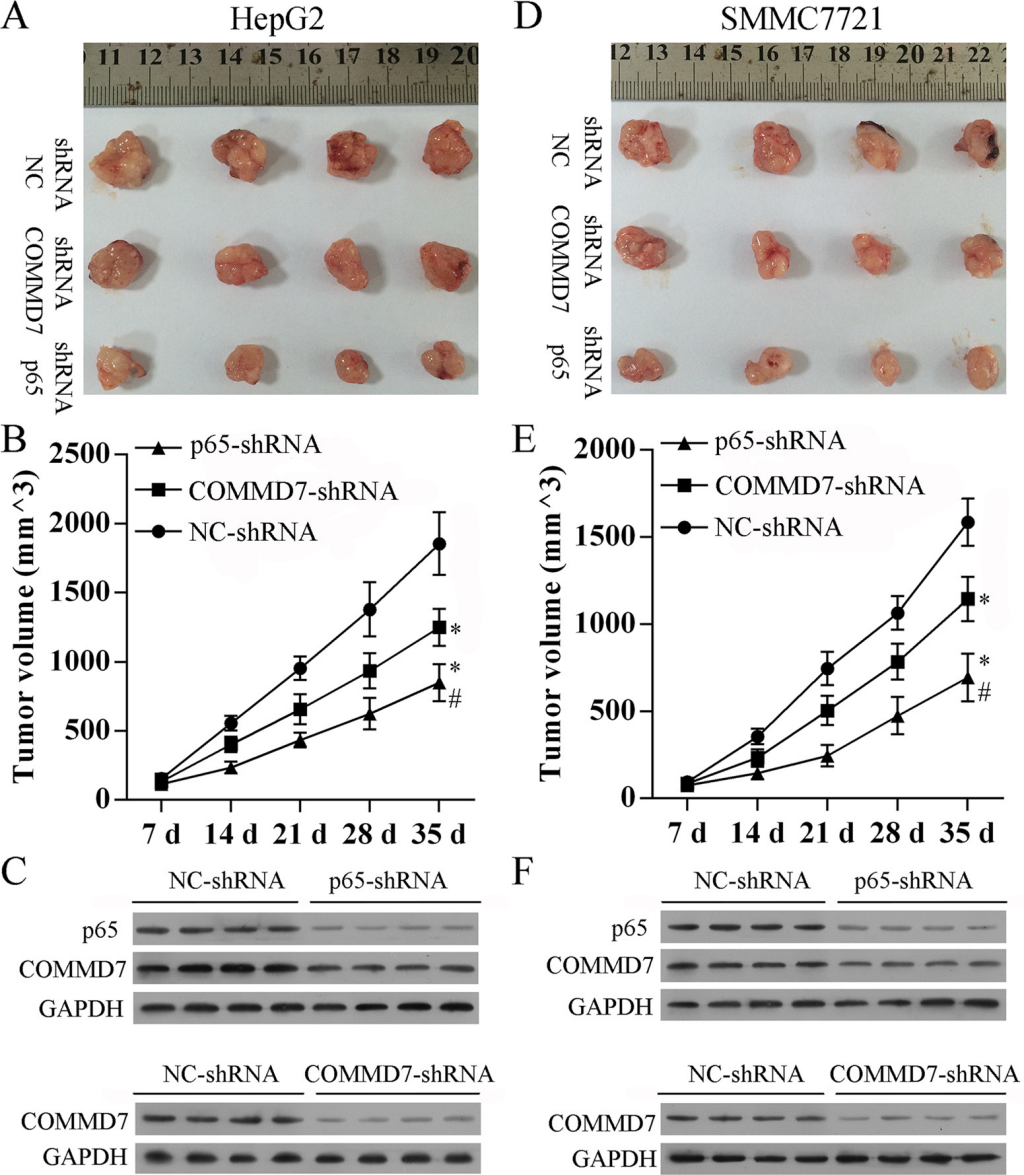
Stable transfection of NF-κB shRNA or *COMMD7* shRNA suppressed tumorigenicity *in vivo* Tumor volume (V) was measured by caliper daily and calculated using the formula V = (L × W^2^)/2, where L was the length and W was the width of the tumor. Growth curves were plotted using average tumor volume within each experimental group every week after respective inoculation of HepG2 cells (**A**–**C**) or SMMC-7721 cells (**D**–**F**) that were stable transfected with *COMMD7* shRNA or NF-κB shRNA, and control cells. Five weeks later, the mice were euthanized, and the dissected tumors were collected (A and D), the tumor growth curves (B and E), and the expression of *p65* or *COMMD7* in tumors (C and F) were performed. *p < 0.05, vs. NC-shRNA; ^#^*p* < 0.05, vs. *COMMD7*-shRNA.

To confirm the above findings, an *in vivo* tumor model was used. Stable transfected HepG2 (Figure 7A–7C), SMMC-7721 (Figure 7D–7F) and control cells were injected separately into nude mice. Similar results were found in HepG2 and SMMC-7721 cells. Five weeks after injection, the group with *COMMD7* shRNA formed substantially smaller tumors than the control group (Figure 7A, 7B, 7D, and 7E). The expression of *COMMD7* in tumors in the group with *COMMD7* shRNA was lower than that in the control group (Figure 7C and 7F). The group with NF-κB shRNA formed substantially smaller tumors than the group with *COMMD7* shRNA (Figure 7A, 7B, 7D, and 7E). The expression of *p65* in tumors in the group with *COMMD7* shRNA was lower than that in the control group (Figure 7C and 7F). Furthermore, *COMMD7* is downregulated in tumors in the group with NF-κB shRNA (Figure 7C and 7F), suggesting the expression of *COMMD7* is correlated with the NF-κB level.

## DISCUSSION

NF-κB and *COMMD7* transcriptional activity appear to play important roles in driving the progression of hepatocellular carcinoma (HCC), especially in tumor progression [9, 18, 19]. It was demonstrated that *COMMD7* (previously named BC047440) is highly expressed in cytoplasm of HCC tissues and human HCC cell lines, while being scarcely expressed in adjacent tissues and normal liver tissue and LO2 cells [10, 20]. Interestingly, our data have shown that *COMMD7* is overexpressed in HCC with a correlation to NF-κB. *COMMD7* was also aberrantly overexpressed in human HCC cell lines, but was significantly less expressed in human liver cell line HL7702.

In previous studies, it was demonstrated that *COMMD7* has an inhibitory role on NF-κB signaling [8, 21]. *COMMD7* binds to IKK complex through NEMO in the nucleus and induced degradation of *p65* leading to the termination of NF-κB signaling [8]. Silenced *COMMD7* expression in HEK293T cells induced a nuclear accumulation of *p65* and a persistent activation of NF-kB upon a continuous TNFα activation [8]. This inconsistent may due to different cell types or cytokine stimulation. Knockdown of *COMMD7* significantly and specifically decreases the *in vitro* proliferation of viable HepG2 cells [10, 11]. In *COMMD7*-silenced HepG2 cells, we observed the impairment of the nuclear translocation of NF-κB by EMSA assay, and the inhibition (75%) of NF-κB using luciferase reporter assay [10]. These findings suggest that *COMMD7* is correlated with the nuclear translocation of NF-κB and the consequent gene transcriptions involved in HCC growth.

We demonstrated that NF-κB directly binds with *COMMD7* promoter and serves as an activator for *COMMD7* transcription, providing a novel molecular mechanism by which NF-κB stimulates transcription of the *COMMD7* gene. This is also supported by the inhibition of NF-κB by knocking down of *COMMD7* [10]. Thus, NF-κB correlated or is correlated with *COMMD7*.

The silencing of *COMMD7* inhibited human HepG2 cell growth showing an anti-proliferative effect [11]. We also showed that cell proliferation was inhibited by *COMMD7* silencing, and NF-κB silencing inhibited the expression of *COMMD7* and further attenuated cell proliferation in both HepG2 and SMMC-7721 cells. Cell apoptosis was promoted by *COMMD7* silencing, and NF-κB silencing further promoted apoptosis. Cell migration and invasion were also inhibited by *COMMD7* silencing, and NF-κB silencing further inhibited the cell migration and invasion. Although the detailed molecular mechanism underlying the anti-proliferative and anti-invasion effect by *COMMD7* silencing is unknown, our findings suggested that it was positively correlated with NF-κB. It has partly revealed the specific target genes and mechanisms of transcriptional activation in these processes. Further studies are required to determine co-activation that mediate this effect.

Taken together with our previous study [22], we have now identified an important mechanism of crosstalk between NF-κB and *COMMD7* that serves to increase the activity of both pathways and drive HCC progression. *COMMD7* activated NF-κB signaling through TRAF6 [22] and silencing of *COMMD7* impaired the TNF-α-activated NF-κB signaling [11], showing an anti-proliferative effect. On the other hand, silencing of NF-κB reduced the expression of *COMMD7*, also showing anti-proliferative, pro-apoptotic, and anti-invasion effects. In addition, the overexprsssion of NF-κB induced an increase of *COMMD7* in both HepG2 and SMMC-7721 cells (Supplementary Figure S1). This suggests that *COMMD7* is correlated with a novel NF-κB positive feedback loop in hepatocellular carcinoma. These results suggest that the therapeutic strategies for HCC should be explored keeping in mind the correlation between NF-κB and *COMMD7*, so as to improve the specificity and sensitivity of therapy and to reduce toxicity.

## MATERIALS AND METHODS

### Ethics statement

Investigation has been conducted in accordance with the ethical standards and according to the Declaration of Helsinki and according to national and international guidelines and has been approved by the Xinqiao Hospital of Third Military Medical University review board. Written informed consent was obtained for all patient samples. Animal experiments were approved by the Institutional Committee for Animal Research and were performed in conformity with national guidelines for the care and use of laboratory animals.

### Clinical samples

Frozen and paraffin-embedded hepatocellular carcinoma, and patient-matched para-carcinoma tissue samples were obtained from Chinese patients who underwent curative resection in the Xinqiao Hospital of Third Military Medical University. No cancer cells were detected at the resection margin. None of the patients had received chemotherapy or radiotherapy before surgery. All specimens were confirmed by pathological examinations. Clinical staging was performed according to the International Union for Cancer Control (UICC). The clinicopathological data is summarized as follows. Patients (10 Males and 10 Females) ages ranged from 41.6 years to 63.8 years (median age: 54.5 yrs). Time from diagnosis to surgery ranged from 9 to 22 days (median: 14 days). The histological grades of primary hepatocellular carcinoma are stage II and III. The tissue samples were frozen in liquid nitrogen and stored at −80°C until the measurement of expression of *COMMD7*.

### Immunohistochemistry (IHC) and immunofluorescence (IFC)

Paraformaldehyde (4%)-fixed and paraffin-embedded tissues were cut into 3-μm-thick sections, de-waxed in xylene and then progressively rehydrated by gradient concentrations of ethanol. Antigens were retrieved by heating the tissue sections at 100°C for 30 min in citrate solution (10 mmol/L, pH 6.0). The sections were cooled and immersed in methanol in the presence of 0.3% hydrogen peroxide for 15 min to block the endogenous peroxidase activity. The sections were subsequently rinsed in PBS for 5 min and then incubated with primary antibody against *COMMD7* (1:150, Abcam) or *p65* (1:100, Abcam) at 4°C overnight. Sections incubated without the primary antibody were used as negative controls. The sections were then incubated with horseradish peroxidase-labeled goat against mouse/rabbit secondary antibody (Maixin Biotechnology, Fuzhou, China). Diaminobenzene or DAPI was used as the chromogen, and hematoxylin was used as the nuclear counterstain.

For immunofluorescence, cell were fixed in 4% paraformaldehyde, followed by a wash in PBS for 5 min. Then, cells were incubated with primary antibody against *COMMD7* or *p65* at 4°C overnight. Cells incubated without the primary antibody were used as negative controls. Cells were then incubated with AF594-labeled goat against mouse/rabbit secondary antibody (Maixin Biotechnology, Fuzhou, China). DAPI was used to label the nucleus. Images were acquired by an FV10i confocal microscope (OLYMPUS, Japan).

### Cell culture

Human hepatocellular carcinoma cell lines including HepG2, SMMC-7721, Huh7 and Hep3B, and human liver cells HL7702 were purchased from Cell Bank of Shanghai Institute of Biochemistry & Cell Biology, Chinese Academy of Sciences. Cells were cultured in RPMI-1640 medium (Invitrogen, USA) and supplemented with 10% heat-inactivated fetal bovine serum, 1% penicillin/streptomycin at 37°C in 5% CO_2_.

### qRT-PCR

Total RNA was isolated using TRIZOL (Invitrogen Inc., USA). RNA concentration was measured by GeneQuant II (Pharmacia, Uppsala, Sweden) at 260 nm. Reverse transcription reaction and cDNA synthesis was performed according to the manufacturer's instructions (Invitrogen). PCR analysis was performed on Applied Biosystems 7500 Sequence Detection system (ABI, USA) using SYBR Premix Ex Taq GC kit (Takara, Japan). The primers for *COMMD7*: 5′-AGTGGCTTTCTCCTCACTAAGACC-3′(forward) and 5′-GGAAAGATTTCTGGCTCAGCTC-3′ (reverse); for *p65*: 5′-CGAACTGTTCCCCCTCATCTT-3′ (forward) and 5′-CTTGGGCTGCTCAATGATCTC-3′ (reverse); for GAPDH: 5′-ACACCCACTCCTCCACCTTT-3′ (forward) and 5′- TTACTCCTTGGAGGCCATGT-3′ (reverse). Gene expression of *COMMD7* and *p65* was normalized to the level of GAPDH within each sample using the relative _ΔΔ_CT method, respectively. Gene expression is shown as relative expression to control. The correlation between *COMMD7* and the expression of *p65* was tested by correlation analysis. The data shown is representative of three independent experiments.

### Western blotting

Samples were lysed on ice for 10 min in 50 mM Tris-HCl (pH 7.5), 10% glycerol, 2% SDS, 0.1 M dithiothreitol (DTT) and 10 mM phenylmethylsulfonyl fluoride (PMSF). Proteins were separated on 10% SDS-polyacrylamide gels and electroblotted onto a nitrocellulose membrane in 25 mM Tris base and 190 mM glycine at 50 V for 3 h at 4°C. To detect the expression of *COMMD7* and *p65*, blots were incubated with monoclonal antibodies against *COMMD7* and *p65* at a concentration of 1:1000, respectively. All antibodies were purchased from Santa Cruz Biotechnology, USA. Proteins were detected by enhanced chemiluminescence (ECL) as described by the manufacturer (Beyotime, China).

### Luciferase reporter assay and transfections

The transcription factor binding sites (TFBS) were identified by Genomatix. pGL4.12-Luc vector (Promega) including the indicated cloned genomic regions and thymidine kinase (TK) promoter was transfected into 293T cells. A Renilla luciferase reporter pRL-TK (Promega) was co-transfected as a control for evaluating transfection efficiency. Transfections were performed with Lipofectamine 2000 or LTX (Invitrogen) according to the manufacturer's instructions. After 24 h, cells were washed before measuring firefly and Renilla luciferase activities using the Dual-Glo^™^ Luciferase Assay System (Promega). In each experiment, firefly luciferase activity was normalized to Renilla luciferase activity.

### Chromatin immunoprecipitation (ChIP)

Cells were cross-linked with 1% formaldehyde for 10 min at RT, followed by addition of glycine to a final concentration of 0.125 M. Next, cells were washed with PBS, collected, washed with cell lysis buffer (5 mM PIPES pH 8.0, 85 mM KCl, 0.5% NP-40, and 10 μg of PMSF/ml) containing protease inhibitors, and resuspended in nuclear lysis buffer (50 mM Tris–HCl pH 8.1, 10 mM EDTA, 1% SDS, and 10 μg of PMSF/ml) containing protease inhibitors. Chromatin was sheared to approximately 200–1000 bp by sonication, prior to a 1/5 dilution in ChIP dilution buffer (16.7 mM Tris–HCl pH 8.1, 167 mM NaCl,1.2 mMEDTA, 0.01% SDS, 1.1% Triton ×−100, and 10 μg of PMSF/ml) containing protease inhibitors. Then, the chromatin solution was incubated with the indicated antibody and Dynabeads^®^ M-280 sheep anti-rabbit IgG (Dynal Biotech) overnight at 4°C. Beads were washed three times with ChIP wash buffer (50mMHEPES–KOH (pH 7.0), 0.5 MLiCl, 1 mMEDTA, 0.7% sodium deoxycholate, and 1% NP-40). Immunocomplexes were eluted from beads using ChIP elution buffer (50 mM Tris–HCl (pH 8.0), 10 mM EDTA, and 1% SDS) for 1 h at 65°C. Elutes were then incubated overnight at 65°C to reverse cross-linking, prior to the addition of 0.5 mg of protease K/ml for 2 h at 55°C. DNA was purified using the MinElute PCR purification kit (QIAGEN) and analyzed by PCR. The primer sequences used for ChIP PCR analysis were shown in Supplementary Table S1.

### Electrophoretic mobility shift assay (EMSA)

DNA–protein binding was assayed with DNA probes that had been 32P-labeled by end-filling with Klenow fragment and NF-κB in the presence or absence of anti-NF-κB antibody or specific/mutant competitors. Anti-NF-κB antibody or specific mutant competitors were pre-incubated with NF-κB for 30 min at 25°C before addition of radiolabeled probes. Reactions were performed in binding buffer [20 mM HEPES (pH 7.9), 10% glycerol, 50 mM KCl, 0.05% NP-40, 0.5 mM EDTA, 0.5 mM DTT, and 1 mM PMSF] for 30 min at 25°C. Products of the binding reactions were separated by polyacrylamide gel electrophoresis (PAGE) on a 4% gel for 3 h at 100 V. The probes used for EMSA assay were shown in Supplementary Table S2.

### Cell transfection

For knockdown of NF-κB or *COMMD7*, the *p65* shRNA, *COMMD7* shRNA and negative shRNA (50 nM) were synthesized and purified by OriGene (OriGene, Rockville, USA). All the sequences for shRNA were shown in Supplementary Table S3. HepG2 or SMMC-7721 cells were seeded in a 6-well plate (2 × 10^5^/well) for 24 h before transfection, and then were transfected with *p65* shRNA, *COMMD7* shRNA or negative shRNA using FuGENE^®^ HD transfection reagent (Promega, USA) in accordance with the manufacture's instruction. Following transfection, cells were selected in G418, and resistant colonies were propagated and examined for the downregulation of NF-κB or *COMMD7* by qRT-PCR, immunofluorescence and western blotting, and cells were used for further experiments.

### Cell proliferation assay

The cell survival of HepG2 and SMMC-7721 cells was evaluated using the MTT [3-(4,5-dimethyl-thiazol-2-y1) 2,5-diphenyl tetrazolium bromide] (Sigma, USA) colorimetric assay, respectively. Cells were seeded in 96-well tissue culture plates at 2 × 10^4^ cells per well for 0, 24, 48, 72, and 96 h. Then, HepG2 or SMMC-7721 cells were washed with PBS and incubated in 100 μL of 5 mg/mL MTT solution (Invitrogen Inc., USA) for 3 h. MTT is converted into purple colored formazen in living cells which was then solubilized with dimethylsulfoxide (DMSO) (Invitrogen Inc., USA) and absorbance of solution was read at 450 nm using a microplate reader Thermo Plate (Rayto Life and Analytical Science C. Ltd, Germany).

### Cell apoptosis assay

Cell apoptosis was determined by Annexin V assay. Following transfection for 48 h, cells were harvested by trypsinization and washed with PBS, and suspended in Annexin V binding buffer. FITC-conjugated Annexin V and propidium iodide (PI; KeyGen, China) were added to the cells successively. After incubation, Annexin V binding buffer was added, and cells were analyzed by a FACScan (Becton-Dickinson, USA) flow cytometer. Annexin V(+)/P(−) and Annexin V(+)/P(+) represent cells in early apoptosis and late apoptosis/necrosis, respectively. Cell apoptosis was further confirmed by Hoechest staining.

### Cell invasion assay

Cell invasion of HepG2 and SMMC-7721 cells were assayed using a transwell chamber (Millipore, USA). Transwell chamber was coated with 30 μl Matrigel and was placed into 24-well plate and incubated for 30 minutes at 37°C. After 48 h of transfection, HepG2 or SMMC-7721 cells were trypsinized and seeded in chambers at the density of 8 × 10^4^ cells per well and cultured in RPMI 1640 medium with 2% serum, while 600 μl of 10% FBS–RPMI 1640 was added to the lower chamber. Twenty-four hours later, migrated cells were fixed with 100% methanol for 30 min and stained using crystal violet for 20 min. Non-migrated cells were removed using cotton swabs. Cell images were obtained under a phase-contrast microscope (Olympus, Tokyo, Japan).

### Tumorigenicity assay in nude mice

Six-week-old female Balb/c athymic nude mice (Vitalriver Laboratory Animals, Beijing, China) were subcutaneously injected in the right flank with 2 × 10^6^ cells in 0.1 mL PBS. Once tumors were formed, tumor volume (V) was measured by caliper daily and calculated using the formula V = (L × W^2^)/2, where L was the length and W was the width of the tumor. The mice were randomly divided into four groups (*n* = 6) for respective inoculation of SMMC-7721 cells or HEPG2 cells that were stable transfected with *COMMD7* shRNA or NF-κB shRNA, and control cells for 42 days after tumors reached an average 32–65 mm^3^. Growth curves were plotted using average tumor volume within each experimental group every week. Five weeks later, the mice were euthanized, and the dissected tumors were collected and prepared for subsequent analyses. All animal experiments were approved by the institutional animal center.

### Statistical analysis

For quantitative data, all results are expressed as the mean ± SD. Statistical significance between groups was determined using one-way analysis of variance (ANOVA) or an unpaired Student's *t*-test using SPSS 18.0 (SPSS, USA). Each experiment was repeated at least three times. *p* < 0.05 was considered statistically significant.

### Ethics statement

Investigation has been conducted in accordance with the ethical standards and according to the Declaration of Helsinki and according to national and international guidelines and has been approved by the Xinqiao Hospital of Third Military Medical University review board. Written informed consent was obtained for all patient samples. Animal experiments were approved by the Institutional Committee for Animal Research and were performed in conformity with national guidelines for the care and use of laboratory animals.

## SUPPLEMENTARY FIGURES AND TABLES


