# Supplementary material for: COMMD7 is correlated with a novel NF-κB positive feedback loop in hepatocellular carcinoma
Source: Oncotarget. 2016 Apr 27;7(22):32774–84. doi: 10.18632/oncotarget.9047 (PMC5078050; doi:10.18632/oncotarget.9047)
Supplement: Supplementary file 1 [file oncotarget-07-32774-s001.pdf]

COMMD7 is correlated with a novel NF-κB positive feedback loop in hepatocellular carcinoma

Supplementary Materials

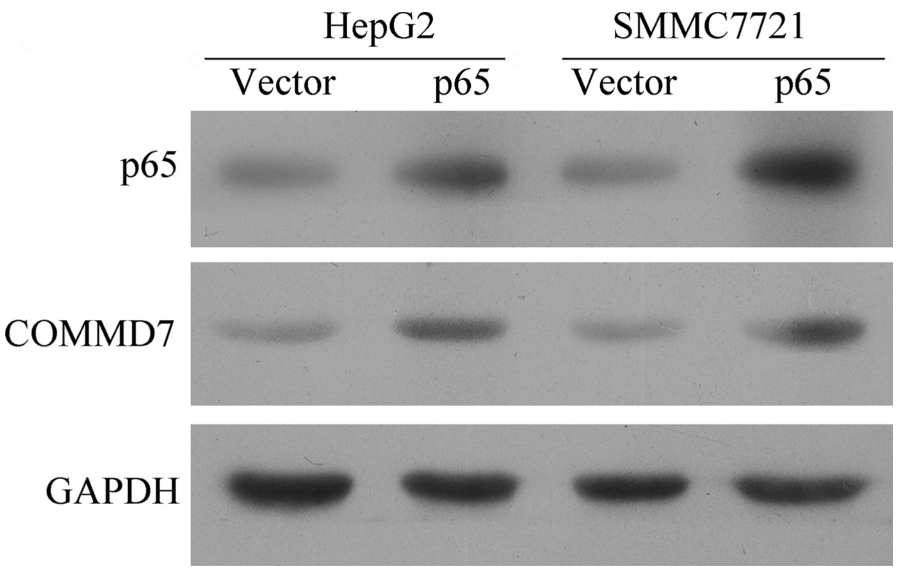

Supplementary Figure S1: Overexprsssion of NF-κB induced an increase of COMMD7 in both HepG2 and SMMC-7721 cells. After transfected with p65-overexpression plasmid, the expression of p65 and COMMD7 in cells were detected.

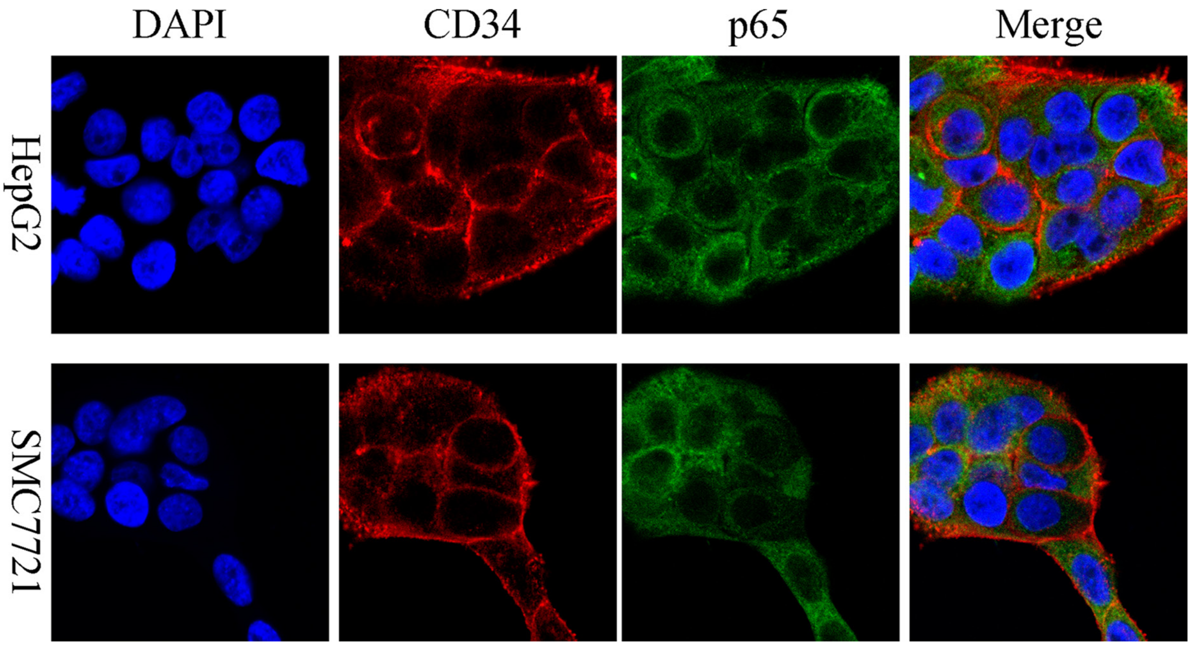

Supplementary Figure S2: The identification of cell types (HepG2 and SMMC7721) by double staining with CD34 and p65.

Supplementary Table S1: Primer sequences used for ChIP PCR analysis

		Primer sequence (5′-3′)
MUT 1	Forward	ATCCGCCCGCCTCAGCCT
	Reverse	CTCTACCCTTTCCTGAAAATCC
MUT 4	Forward	TCTCTATTTTCTCATGTTTCC
	Reverse	CTCCTCACAGTTCCCAGGTG
MUT 5	Forward	CCTTCCAAGGTTGAGACCTAG
	Reverse	TGCCAGACACTGGGGACA
MUT 6	Forward	CTGGCCTGCTTTGTTCCCTTC
	Reverse	ATATGGCTAGCTGCTATTATCATC

Supplementary Table S2: Probes used for EMSA assay

Name	Sequence (5′-3′’)
MUT 1	^{32}P-labeled-GTGCTGGGATTACAGGAGTG
MUT 4	^{32}P-labeled-CCAGTGGGGATTAGGGTAATATTC
MUT 5	^{32}P-labeled-CTCTGGCCTGCTTTGTTCCCTTC
MUT 6	^{32}P-labeled-CCTGTCCCCAGTGTCTGGCATCG

Supplementary Table S3: shRNA sequences

Gene		Sequence (5′-3′)	Target sequence
COMMD7	Forward	AUCUAUGAGCUGGUUAAUCAU	ATGATTAACCAGCTCATAGATAT
	Reverse	GAUUAACCAGCUCAUAGAUAU	
p65	Forward	UUACGUUUCUCCUCAAUCCGG	CCGGATTGAGGAGAAACGTAAAA
	Reverse	GGAUUGAGGAGAAACGUAAAA	
